# Neural underpinnings of ethical decisions in life and death dilemmas in naïve and expert firefighters

**DOI:** 10.1038/s41598-024-63469-y

**Published:** 2024-06-08

**Authors:** Isabel C. Duarte, Ana Dionísio, Joana Oliveira, Marco Simões, Rita Correia, Joana A. Dias, Salomé Caldeira, João Redondo, Miguel Castelo-Branco

**Affiliations:** 1 Institute for Nuclear Sciences Applied to Health (ICNAS), Coimbra Institute for Biomedical Imaging and Translational Research (CIBIT), University of Coimbra, Coimbra, Portugal; 2 Faculty of Medicine (FMUC), University of Coimbra, Coimbra, Portugal; 3 Faculty of Science and Technology, Center for Informatics and Systems of University of Coimbra (CISUC), University of Coimbra, Coimbra, Portugal; 4 Centre for Prevention and Treatment of Psychological Trauma (CPTTP), Department of Psychiatry, Coimbra University Hospital Centre (CHUC), Coimbra, Portugal

**Keywords:** Human behaviour, Decision

## Abstract

When a single choice impacts on life outcomes, faculties to make ethical judgments come into play. Here we studied decisions in a real-life setting involving life-and-death outcomes that affect others and the decision-maker as well. We chose a genuine situation where prior training and expertise play a role: firefighting in life-threatening situations. By studying the neural correlates of dilemmas involving life-saving decisions, using realistic firefighting situations, allowed us to go beyond previously used hypothetical dilemmas, while addressing the role of expertise and the use of coping strategies (n = 47). We asked the question whether the neural underpinnings of deontologically based decisions are affected by expertise. These realistic life-saving dilemmas activate the same core reward and affective processing network, in particular the ventromedial prefrontal cortex, nucleus accumbens and amygdala, irrespective of prior expertise, thereby supporting general domain theories of ethical decision-making. We found that brain activity in the hippocampus and insula parametrically increased as the risk increased. Connectivity analysis showed a larger directed influence of the insula on circuits related to action selection in non-experts, which were slower than experts in non rescuing decisions. Relative neural activity related to the decision to rescue or not, in the caudate nucleus, insula and anterior cingulate cortex was negatively associated with coping strategies, in experts (firefighters) suggesting practice-based learning. This shows an association between activity and expert-related usage of coping strategies. Expertise enables salience network activation as a function of behavioural coping dimensions, with a distinct connectivity profile when facing life-rescuing dilemmas.

## Introduction

Decision-making processes involve making difficult choices, weighing risks and potential rewards, to act and to evaluate the consequences of our actions^1–3^. Particular types of decisions involving human conflict are moral and ethical decisions, both regarding distinctions either between right or wrong. Both require the evaluation of choices involving outcomes of variable magnitude and probability. A previous functional magnetic resonance imaging (fMRI) study has addressed this issue using hypothetical dilemmas to evaluate the moral acceptability of sacrificing a single life to save a larger group^4^. The main difference when it comes to ethics is that its principles refer to community values more than personal values. A largely known example is the Hippocratic Oath in medicine: naïve individuals are not explicitly aware of deontological norms, which raise the interesting question of whether the neural underpinnings of deontologically based decisions are affected by expertise.

Here we studied decisions in a real-life setting involving life-and-death outcomes that affect others and the decision-maker. We chose a situation entailing a reason/emotion conflict and where expertise can play a role: firefighting in life-threatening situations. Our design comprised critical aspects of moral/ethical decision^4^: trade-offs among costs and benefits of varying magnitude; uncertainty, with outcomes that vary in their probability; and life-and-death outcomes which were also directly self-relevant, a unique feature of our paradigm regarding previously reported ethical dilemmas. Our study does depart from hypothetical life-and-death moral dilemmas whereby one can save several lives by sacrificing a smaller number of lives^5–7^. The present study also uses a parametric design of risk probability to study the neural representation of contextual salience.

Firefighters face unique challenges requiring decision-making, which often leads to highly conflicting dilemmas because of alternatives that may be largely unfavourable. They often operate in high-stress, time-constraining situations where decisions must be made quickly and accurately to ensure the safety of themselves and others^8^. They receive dedicated training under these conditions and rely on their experience to make decisions. However, in spite of the potential advantages posed by expertise, exposure to trauma or highly stressful situations may also impair cognitive processes such as working memory, attention, and executive functioning, which directly affects decision-making^9,10^. It is well known that firefighters are a population at special risk of developing post-traumatic stress disorder and other stress-related disorders^11^. In fact, in a recent study, a global prevalence of 8.6% possible post-traumatic stress disorder and 14.4% of possible psychopathology was found in a sample of 139 firefighters^12^. This implies that coping abilities are continuously challenged which might potentially impact on decision-making.

Coping abilities refer to the cognitive and behavioural strategies that individuals use to manage stressors and regulate their emotions^13^. The use of coping strategies by firefighters can reduce overwhelming feelings of anxiety and help make effective decisions in demanding situations. Examples of coping strategies include seeking instrumental support (asking for advice or help from others) and emotional support (searching for emotional support and understanding from others)^14^.

Neuroimaging studies have revealed a network of brain regions that are involved in value-based decision-making tasks, including the prefrontal cortex, anterior cingulate cortex, insula, and the striatum^15–17^. In particular, when the risk varies, the ventromedial prefrontal cortex has been implicated in evaluating the expected value of different options^18–20^, thereby contributing to the implementation of adaptive behaviours^21^. The anterior cingulate cortex and insula are thought to play a role in processing conflict and emotional and affective aspects of decision-making^22,23^. Both areas form the core nodes of the salience network while interacting with the amygdala, being responsive to important changes in the environment, in particular unsafe stimuli and contexts^24,25^. As part of the salience network, the anterior insula represents a hub in decision-making and behavioural guidance^26–28^. It is involved in decision under uncertainty which is critical for generation of adaptive behavioural responses^29^ during error monitoring^30,31^. Previous studies using decision-making tasks requiring interpretation of social cues and regulation of social behaviour show a role for anterior insula in emotional processing and social cognition^32,33^. The striatum, in particular its ventral subdivisions, is involved in reward processing, based on prediction error signals and plays a critical role in guiding behaviour based on expected outcomes^34–36^. While much of the research on decision-making has focused on neuroeconomic paradigms, other studies have demonstrated similar brain activation patterns in response to non-monetary risks, such as those involving social or health-related decisions^22,37,38^. Concerning moral or ethical decisions it is believed that a similar domain-general valuation mechanism might be operating^4,25^.

In this study, we investigate the decision-making mechanisms involving ethical decisions and the role of expertise using a self-involved ethical task that mimics a genuine life-threatening rescuing scenario in a burning house. This was achieved by using fMRI in a cohort of experienced firefighters and non-firefighters. We asked whether expertise would influence the neural correlates of ethical decision-making. We also explored the impact of expertise on effective connectivity and finally whether coping strategies influence the neurobehavioral patterns.

## Methods

### Sample characterization

In this experiment, we included a general sample of 47 volunteers, subdivided into two groups of firefighters and non-firefighters. The total cohort was composed of seventeen females and 30 males, the mean age was 32.30 ± 9.12 (mean ± SD), the mean for years of education was 14.77 ± 2.25, one participant was ambidextrous while the rest were right handed, as assessed using the Edinburgh Handedness Inventory^39^. The final sample included twenty firefighters and 27 non-firefighters.

Firefighters and non-firefighters did not differ in gender (χ^2^ = 3.943, *p* = 0.067) or handedness (*U* = 319.0, p = 0.279), with a marginal age difference (34.85 ± 8.55 and 30.50 ± 9.22 years, respectively, *U* = 178.5, p = 0.05) and education level (14.05 ± 1.82 and 15.30 ± 2.42 years of education, respectively, *U* = 363.5, *p* = 0.036). The two groups, firefighters and non-firefighters, were matched considering the presence and severity of post-traumatic stress disorder symptoms (7.80 ± 13.21 [0–49] and 4.48 ± 5.35 [0–23] respectively, *U* = 285.0, p = 0.743, assessed using the PTSD Checklist for DSM-5), however, the firefighter cohort presented higher scores for symptoms of psychopathology (0.58 ± 0.48 [0.13–2.25] and 0.37 ± 0.30 [0.04–1.26] respectively, *U* = 175.5, p < 0.042, assessed using the Global Severity Index of Brief Symptom Inventory), as expected from occupational burden. Details about the assessment tools are given in the Psychological Assessment sub-section.

Initially, another three participants were recruited but were excluded due to excessive movement during scanning (> 5 mm), poor image (substantial signal loss due to susceptibility artifact) or dropout before functional scanning.

Inclusion criteria included: being able to give informed consent; being aged between 18 and 75 years; normal visual and auditory sensitivity; no history of clinically relevant neuropsychiatric conditions; not taking antipsychotics; absence of alcohol or drugs addiction; and have no contraindications to MRI. In addition, firefighters must have an active involvement in fire extinction activities and non-firefighters participants must not have been involved in more than one fire event.

All procedures were approved by the Ethics Committees of the Faculty of Medicine of the University of Coimbra and of the Coimbra University Hospital Centre*.* All research was performed in accordance with relevant guidelines/regulations. Research involving human research participants was performed in accordance with the Declaration of Helsinki Written and informed consent was obtained from all participants.

### Experimental task

The experimental task was programmed in Vizard 5 (WorldViz Inc., Santa Barbara, California, USA). The stimuli were presented on a 40-inch LCD monitor, with 485 × 878 mm of active screen and 1920 × 1080 resolution. The distances from the eye to the top and the bottom of the screen were 1750 mm and 1825 mm, respectively.

We adopted a mixed block event-related design with a decision-making paradigm involving a firefighting action. Participants from both groups were instructed to play as if they were called to a fire event and had to act as firefighters. Delegates from local Firefighters’ Corporations were involved in refining the experimental design (the national firefighters’ school “*Escola Nacional de Bombeiros*” in Sintra, “Escola de Bombeiros da Lousã” and the firefighters federation “*Federação de Bombeiros do Distrito de Coimbra”*).

**Figure 1:**
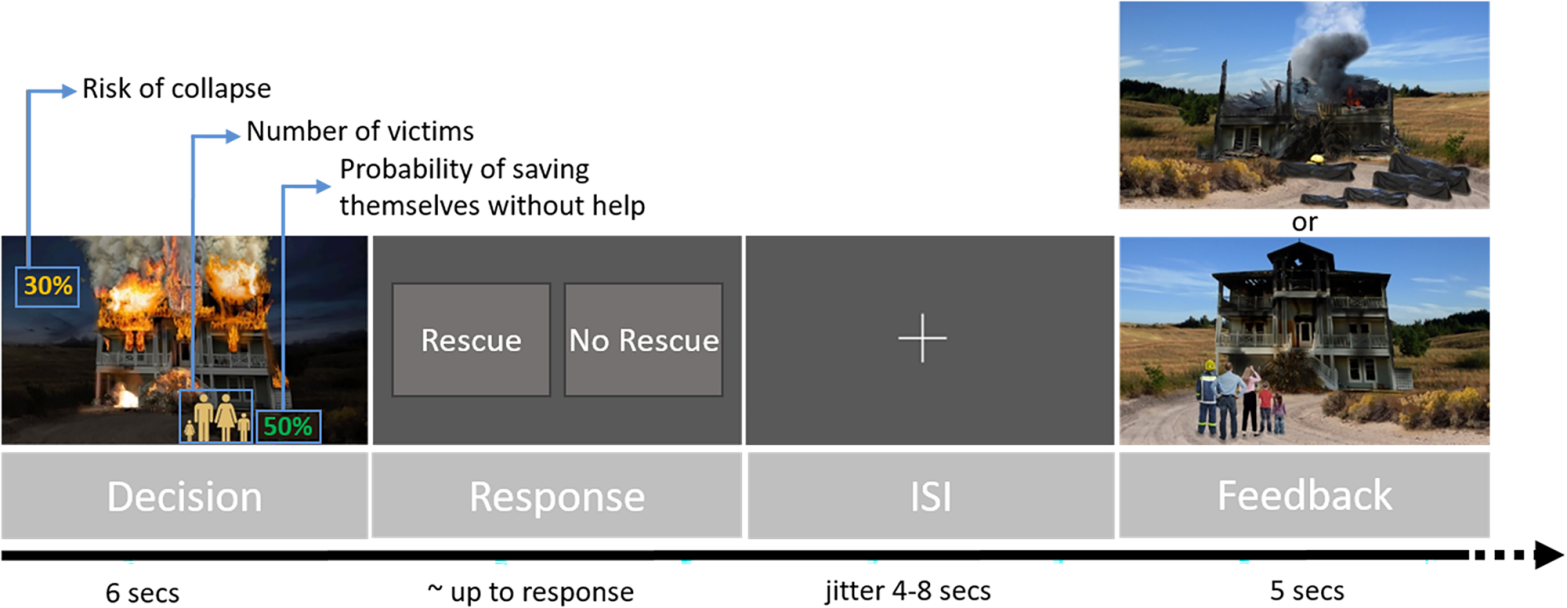
Experimental design. Each trial included a decision phase (6s), a response period and the feedback presentation (5s). In the decision phase, participants saw a burning house, the risk of house collapsing (10–70%), the number of victims (1 or 4), and the probability of the victims escaping without help (10–70%). In the response phase the participant decided between the options of rescue or no rescue. In the case of rescue, his/her own life was also in danger, but the probability to saving the victim(s) if the house did not collapse increased to a fixed value of 80%. The feedback was given showing as outcomes of what happened to the house, the victims and the firefighter character.

We included 3 runs, with 20 trials each, for a total of 60 trials per participant. Each trial included a decision phase, a response period and a feedback presentation. In the decision phase, the initial scenario (Fig. 1) is presented for 6 s. This included a burning house, the number of potential victims inside the house (1 adult or a family of 4), the current risk of the house collapsing (given in % values), and the probability of the victims escaping without external help (given in % values). Both probabilities varied from 10 to 70%, in increasing steps of 20%. The decision involved weighing these probabilities. The participant had to decide, from this information, whether he/she would enter/not enter the house to try rescuing the potential victims. They knew that if the decision was to rescue, their own life was also in danger, but the probability of saving the victim(s) increased (fixed value of 80%). The response was given in a separate block, where the positions of the two possible action (“Rescue” and “No Rescue”) were randomly displayed to account for motor preparation during the decision phase. The duration was set up until the participant’s response. After a variable inter-stimulus interval, the feedback was given, representing what happened to the house, the fate of the potential victims and of the firefighter character (5 s) (Fig. 1). The participants were also asked to give feedback based on the valence and arousal evoked by each trial. The baseline period was jittered between 8 and 12 s. Responses were given through a Hybridmojo MR-compatible joystick.

### Debriefing and sense of presence inventory

After the fMRI session, we asked the participants whether they had adopted a strategy and, if so, whether they adjusted that strategy at some point in the experiment. The majority of the participants (87.2%) adopted a strategy to help decide whether they would enter or not in the house, among which 57.5% adjusted the strategy at some point in the experiment. Furthermore, participants rated how much the experience was emotional, personal and intense on a 5-point Likert scale (where 1 indicated strongly disagree and 5 strongly agree). Our task was overall classified by the participants as emotional (median 4.00 [inter quartile range (IQR) 1]). “Personal” ratings had a median of 3.00 [IQR 2] and intensity, 3.00 [IQR 1]. Debriefing results revealed no differences between firefighters and non-firefighters (*p* > 0.21).

We used the Sense of Presence Inventory^40,41^. To assess the involvement of each participant in the task considering the factors Engagement and Ecological Validity. Each question was rated on a 5-point Likert scale, where 1 corresponded to strongly disagree and 5 to strongly agree. Our experimental task was rated with a median of 3.33 [IQR 0.83] for engagement and 3.20 [IQR 1.20] for ecological validity. Results for the median values were similar in both groups (Engagement: *U* = 219.5, *p* = 0.275; Ecological Validity: *U* = 208.5, *p* = 0.183).

### Magnetic resonance imaging

Brain images were obtained from a 3T Siemens MAGNETOM Prisma Fit scanner (Siemens, Erlangen) using a 64-channel birdcage head coil. We acquired a magnetization‐prepared two rapid gradient echo sequence to obtain anatomical images, with the following parameters: TR (repetition time) = 5000 ms, TE (echo time) = 3.11 ms, TI_1_ (first inversion time) = 700 ms, TI_2_ (second inversion time) = 2500 ms, FA1 (first flip angle) = 3°, FA2 (second flip angle) = 5°, voxel size = 1 × 1 × 1 mm^3^, FOV (field-of-view) = 256 × 256 mm^2^, 192 slices.

Three runs of functional magnetic resonance imaging were acquired through the multi-band accelerated echo planar imaging sequence. We used phase encoding in the anterior–posterior direction, multi-band acceleration factor 6, a 1000 ms TR, a 37 ms TE, a flip angle of 68°, voxel size of 2 × 2 × 2 mm^3^ and FOV of 200 × 200 mm^2^, covering 72 axial slices. To correct for image distortion, we acquired a short echo planar imaging sequence (10 volumes) with the same parameters but with posterior-anterior phase encoding direction before each functional run.

### Data analysis

MRI data were analysed in BrainVoyager 22.4 software (Brain Innovation, Maastricht, The Netherlands). We denoised the background, applied inhomogeneity correction to the structural data, and normalized images to the Montreal Neurological Institute (MNI) template space. A mask excluding the eyes, scalp and bone was created from an average file of all participants. Functional data pre-processing steps included slice scan time correction (cubic-spline interpolation), 3D head-motion correction (trilinear- and sinc-function-based interpolation) and temporal filtering (Fourier-based General Linear Model (GLM), 3 cycles/time course). Functional runs with motion equal to or above 5 mm or 5 degrees in any axis were rejected. Therefore, we excluded runs from 1 control participant and 5 (firefighters: n = 3 runs; controls: n = 2 runs) due to excessive motion. We also ran the COPE plugin for EPI distortion correction^42^. A 6 mm full-width-at-half-maximum Gaussian kernel was used for spatial smoothing of the volume time course.

We generated GLM predictors by convoluting the boxcar function with a two-gamma HRF (hemodynamic response function) and added motion translation and rotation parameters (z-transformed) as confounders in the model. GLM random effects (RFX) analyses were performed. The statistical maps presented in the "Results" section were calculated at group level and overlaid on a single-subject brain for visualization purposes. We used false discovery rate (FDR), with q(FDR) < 0.01, to correct the statistical maps for multiple comparisons. A minimum cluster size of 24 contiguous voxels was established for correction in addition to the conjunction correction. A parametric predictor for the decision phase was also included to account for the risk of house collapse (%). In this approach, a weight is assigned to each trial according to the risk of collapse for that trial. The parametric analysis is presented in a conjunction analysis between the parametric and the main predictor for the decision condition. As the conjunction analysis effectively diminishes the false positive problem, the map was corrected using a cluster extension method. A minimum cluster size of 24 contiguous voxels was estimated based on Monte Carlo simulations (1000 iterations). This parametric analysis allows a search for modulatory effects (regions in which activity increases with the risk of the house collapsing).

Independent correlation analysis was conducted in defined regions-of-interest (ROI) extracted from the statistical maps presented in the "Results" section. For this, individual beta-values were extracted and correlated with individual coping variables.

Effective connectivity was tested using the complete runs in the Granger Causality Mapping v1.7 plugin for BrainVoyager. We used the insula (functionally defined using the contrast *No-rescue VS Rescue*) as a seed ROI. We further compared firefighters and non-firefighters in a RFX analysis. The resultant map is presented with a fixed *p*-value of 0.01, corrected using the cluster extension method. The minimum cluster size of 57 contiguous voxels was estimated based on Monte Carlo simulations (1000 iterations).

### Psychological assessment

Participants underwent psychological assessment, which included self-report measures of psychopathology and mental health, severity of post-traumatic stress disorder, and coping styles for characterization of the sample. The Brief Symptom Inventory (BSI) ascertains symptoms of psychopathology^43,44^ (internal consistency: 0.62 < α < 0.80). All participants then completed the Life Events Checklist for DSM-5 (LEC-5) to assess exposure to traumatic events in their personal life^45,46^ (internal consistency: 0.77 < α < 0.94). Additionally, firefighters completed a similar questionnaire specific for firefighting work, assessing the amount of exposure and disturbance due to traumatic events^47^. They were requested to consider the most adverse/traumatic event and to respond to the post-traumatic stress disorder Checklist for DSM-5 (PCL-5) which provides a global score reflecting the probable presence and severity of PTSD symptomatology^48^. All participants completed the Brief Coping Orientations to Problems Experienced Inventory (Brief-COPE) to evaluate the magnitude to which participants employed coping strategies in their personal day-to-day lives^49,50^ (internal consistency: 0.55 < α < 0.84), under a dispositional coping style approach, as a habitual stable mechanism that individuals tend to use for dealing with various stressful situations in life^14^. Additionally, the firefighter cohort was asked to complete a second Brief-COPE, referring exclusively to the firefighting activity.

### Statistical analysis

Statistical analysis of the data was carried out in SPSS Statistics 27 (IBM SPSS Statistics, IBM Corporation, Chicago, IL). We used parametric tests for all results presented in the "Results" section. Apart from the fMRI statistical maps, we adopted a significance level of 0.05 for comparison between groups and correlations. To compare times of response, we excluded outlier trials (where participant was slower than his/her mean + 4 × standard deviations). No participant has more than one outlier. Twenty-one participants had one outlier. Comparisons between correlation coefficients were done using the online version of the Cocor package for R^51^. Comparisons between correlation coefficients were only performed when, at least, one of the correlation coefficients was significant. One-sided statistics were used in the comparison between correlations and the *p*-values presented in the results section are FDR corrected.

## Results

### Behavioural data

Considering the experimental design, three variables would hypothetically influence participants’ decision on enter or not to rescue the victims in each trial (risk of collapse, number of victims inside the house and the probability of the victims escaping without help). Considering the behavioural data, participants entered to rescue more often if the victims were a family (entered to rescue 81% ± 12% of those trials) instead of only an individual (entered to rescue 75% ± 14% of those trials) (paired t-test, *t*_(45)_ = 3.888, *p* < 0.001, Cohen’s d = 0.573). Also, the risk of collapse influenced participants’ decisions (repeated measures ANOVA, F_(2.2, 100.8)_ = 99.874, *p* <  < 0.001, partial eta-squared η^2^ = 0.689) (see Supplementary Fig. 1). Post hoc tests show that the participants enter to rescue on fewer trials when the risk of collapse was 70% (the maximum) than when the risk was 50% (*p* <  < 0.001). Similarly, participants enter on fewer trials when the risk was 50% comparing with the risk of 30% (*p* <  < 0.001). Between the most favourable risks, although the participants enter on fewer trials when the risk was 30% than when it was 10%, there was no statistical difference in this particular posthoc test. The probability of the victims escaping without help did not influence participants’ decisions (note that the participants knew that the decision to rescue would always increase the probabilities of saving). Overall, participants decided to enter the house (rescue action) 78% ± 12% of the times (mean ± SD) to try to rescue the potential victims (mean 47 out of 60 trials, minimum of 26 and maximum 59 trials). The victims were effectively rescued 47% ± 7% of the times (mean 28 out of 60 trials, minimum of 19 and maximum 35 trials).

Considering response time, one could expect that the No-rescue decision would take longer. Indeed, the mean response time on the No-rescue decision (2.52 ± 0.86 s) was higher than on the decision to rescue (2.11 ± 0.48 s) (paired t-test, *t*_(45)_ = 3.824, *p* < 0.001, Cohen’s d = − 0.564).

Looking at group comparisons, considering the response times for the decisions No-rescue and Rescue, firefighters and non-firefighters showed to respond differentially (mixed ANOVA, F_(1, 44)_ = 5.888 *p* = 0.019, partial eta-squared η^2^ = 0.118). Post hoc tests showed that firefighters are faster than non-firefighters and this is statistically significant for the No-rescue decision (p = 0.028, eta-squared η^2^ = 0.105), but not significant for the Rescue decision (p = 0.064, eta-squared η^2^ = 0.076). The number of trials where the participant decided to rescue was not different between firefighters and non-firefighters (*t*_(45)_ = − 0.209, *p* = 0.835, Cohen’s d = − 0.062). The percentage of trials where the victims were effectively rescued were not different between firefighters and non-firefighters (*t*_(45)_ = − 1.573, *p* = 0.123, Cohen’s d = − 0.464). The ANOVA analysis, considering both groups and the four levels for the risk of collapse factor, showed no main effect of group (F_(1, 44)_ = 1.828 *p* = 0.183, partial eta-squared η^2^ = 0.040).

### Functional imaging

**Figure 2:**
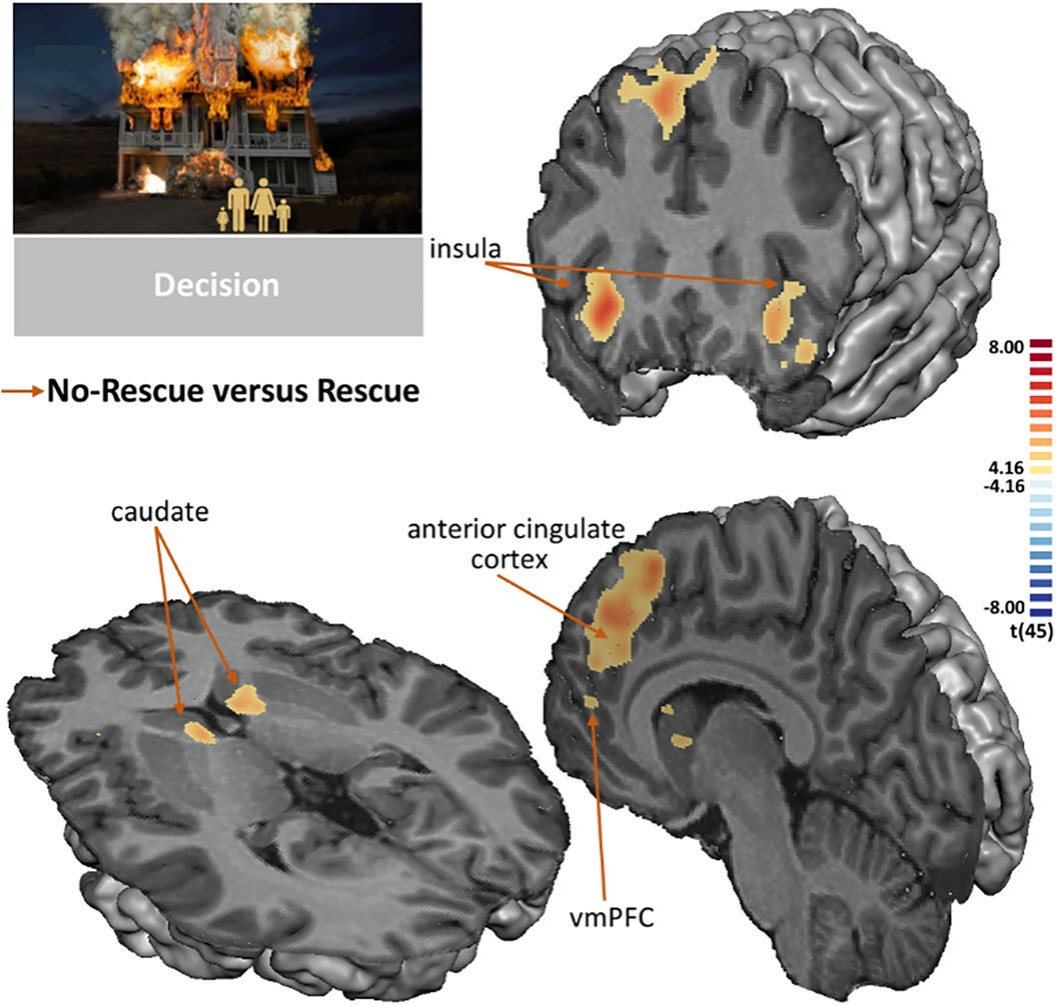
Statistical map of the contrast No-Rescue VS Rescue, during the decision phase (RFX, t_(45)_ > 4.16, p < 0.01, FDR-corrected, minimum cluster size of 24 contiguous voxels). Differences were found in the ventral insula bilaterally, ventromedial frontal gyrus, bilateral caudate head, posterior cingulate cortex, and in a medial frontal cluster that includes the anterior cingulate cortex and the superior frontal gyrus.

During the decision phase, we contrasted the blocks in which the participants decided to refrain from entering the house for a rescue action versus entering to rescue (No-rescue VS Rescue). We found higher BOLD activity in the ventral anterior insula bilaterally, ventromedial prefrontal cortex, bilateral caudate head, posterior cingulate cortex, and in a medial frontal cluster that encompasses the anterior cingulate cortex and the superior frontal gyrus (RFX, t_(45)_ > 4.16, p < 0.01 FDR corrected). The statistical map, highlighting activations within the salience network, is presented in Fig. 2 and details are described in Supplementary Table 1.

**Figure 3:**
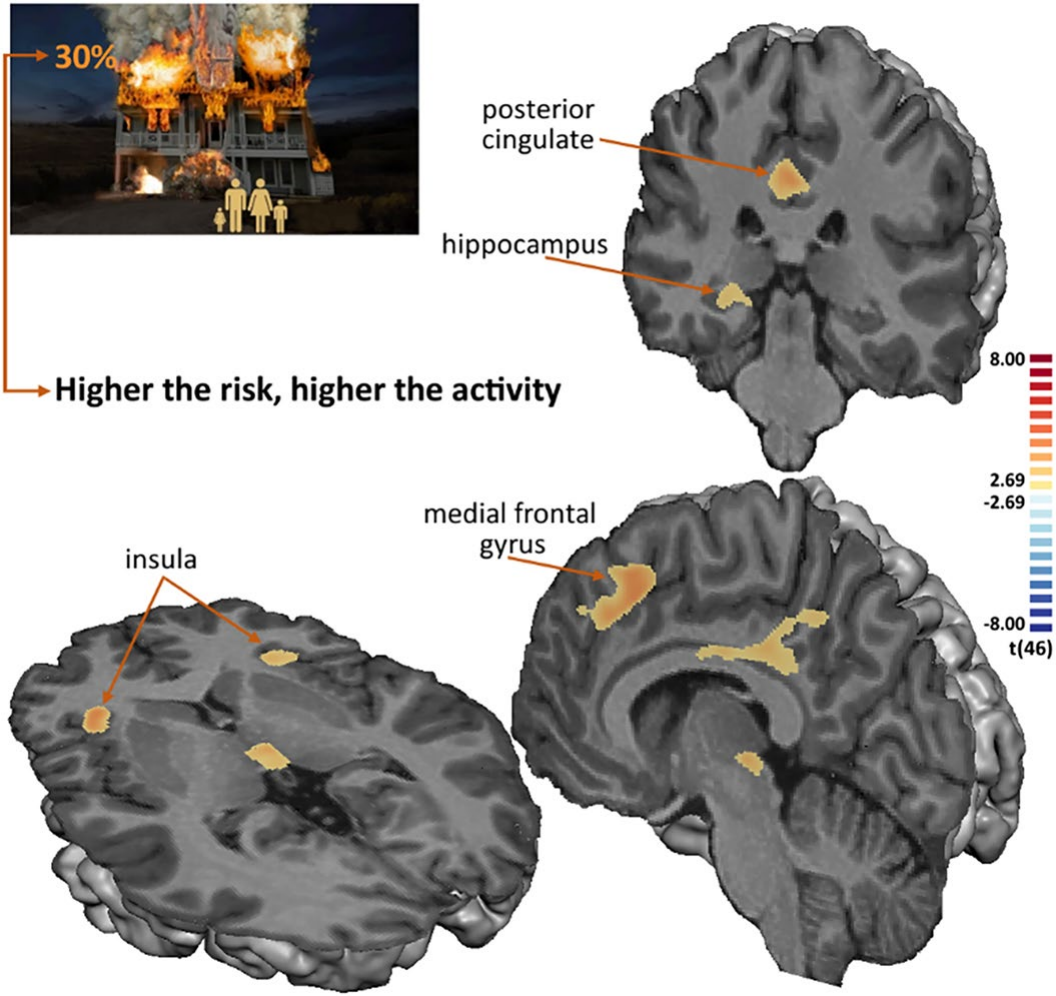
Statistical map of the parametric contrast, in which the decision predictor is modulated by the risk of house collapse (conjunction analysis with the main contrast during the decision phase (RFX, t_(46)_ > 2.69, p < 0.01, minimum cluster size of 24 contiguous voxels). Activity in the right posterior hippocampus and anterior insula increases as the risk of house collapse increases.

Still in the decision phase, we investigated activity changes that were modulated by the risk of house collapse. This parametric analysis allows to identify regions in which activity increases as a function of increases in risk. We found this modulatory effect in the dorsal anterior insula, right hippocampus, medial frontal gyrus, right dorsolateral prefrontal cortex, and posterior cingulate (RFX, t(46) > 2.69, p < 0.01, minimum cluster size of 24 contiguous voxels). The statistical map is presented in Fig. 3 and details are described in Supplementary Table 2.

**Figure 4:**
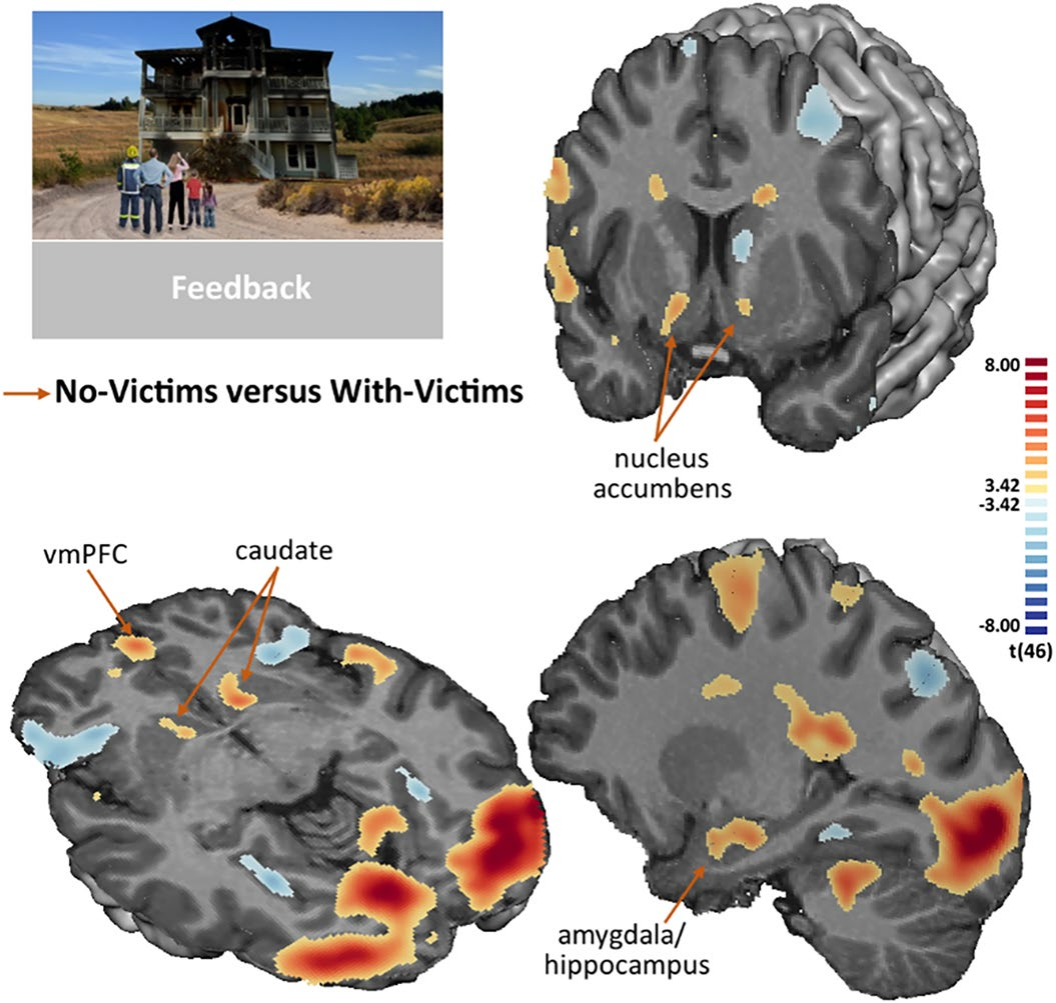
Statistical map of the contrast No-Victims VS With-Victims, during the feedback phase (RFX, -3.42 > t_(46)_ > 3.42, p < 0.01, FDR-corrected, minimum cluster size of 24 contiguous voxels). Differences were found in the nucleus accumbens bilaterally, in the right amygdala and hippocampus bilaterally, ventromedial prefrontal cortex, and cingulate gyrus.

During the feedback phase, we contrasted the blocks in which there were no victims (successful) versus the ones in which there were victims (failed). We found higher activation in reward and limbic regions, namely the nuccleus accumbens bilaterally, the right amygdala and hippocampus bilaterally, ventromedial prefrontal cortex, and cingulate gyrus (RFX, -3.42 > t_(46)_ > 3.42, p < 0.01 FDR corrected). The statistical map is presented in Fig. 4 and details are described in the Supplementary Table 3.

For all these contrasts, the comparison between firefighters and non-firefighters yielded no significant differences suggesting that similar neural networks are recruited for this type of decision irrespective of the level of expertise. The question then emerges if these networks modulate distinctly as a function of coping strategies.

### Correlation analysis with coping strategies

Coping refers to the use of strategies and efforts by individuals to minimise distress and regulate emotions responding to serious and negative circumstances. Comparisons between groups were done for the Brief-COPE questionnaire related to coping strategies in personal day-to-day lives, given that all participants fulfilled this version.

Firefighters are often exposed to highly stressful situations, and they receive dedicated training. Nevertheless, we were able to establish using the Brief-COPE inventory that firefighters did not report different employment of coping strategies when compared to non-firefighters (emotional support: t_(44)_ = − 1.182, p = 0.243, Cohen’s d = − 0.354; instrumental support: t_(44)_ = − 1.045, p = 0.302, Cohen’s d = − 313; self-blame: t_(44)_ = − 0.825, p = 0.414, Cohen’s d = − 0.247; acceptance: t_(43)_ = − 0.173, p = 0.863, Cohen’s d = − 0.053). This still leaves open the question of whether the ability to use coping strategies is linked to task related activity by the two groups in a different way. We investigated correlation patterns between BOLD activity (beta-values) in identified task related brain regions and scores from the Brief-COPE inventory.

**Figure 5:**
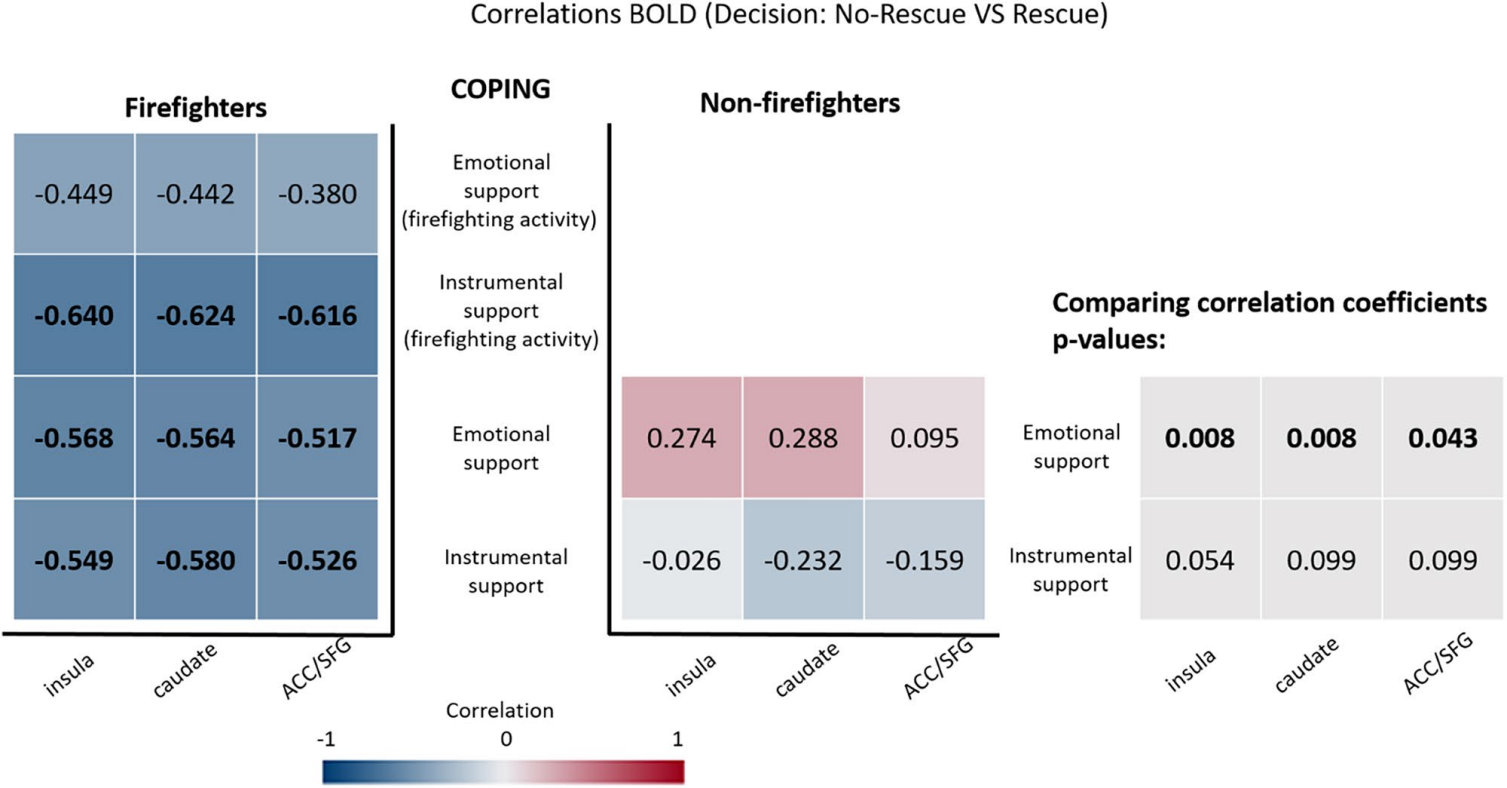
Correlations between individual beta-values from the contrast No-rescue VS Rescue in regions of interest (horizontal axis) and the Emotional and Instrumental strategies of Brief-COPE inventory (vertical axis). Significant correlations are shown in bold. The correlation coefficients are colour coded for visualization purposes (blue for negative and red for positive correlation coefficients). The FDR corrected *p*-values for the comparison of two correlations are presented in the right side and significant values are marked in bold.

Considering the contrast No-rescue VS Rescue, firefighters showed a negative correlation between the beta-values, indexing brain activity, and the emotional support and instrumental support strategies, while non-firefighters did not. This correlation means that the higher the usage of coping strategies, the higher the difference in neural activity between the decision of rescue and the decision of not to rescue. In other words neural activity related to the decision of not rescueing relative to rescuing is associated with lower recruitment of coping strategies in firefighters. The between group comparison of correlation coefficients, after FDR correction, showed that the correlations are different for emotional support in the insula (Fisher's z = − 2.813, p = 0.008 FDR corrected), in the caudate (z = − 2.841, p = 0.008 FDR corrected), and in the anterior cingulate cortex (z = − 2.028, p = 0.043 FDR corrected) (see Fig. 5). Whole-brain correlation maps for the emotional support and instrumental support strategies are also provided as supplementary material (Supplementary Figs. 2 and 3, respectively).

Supplementary Figs. 4–6 show graphical compositions that explore the significant correlations between beta-values from the contrast No-rescue VS Rescue and the Emotional and Instrumental strategies of Brief-COPE inventory in the insula, caudate nucleus and the anterior cingulate cortex. Those results show that the activity in these areas increases in the contrast No-rescue VS Rescue mainly because of the increased activity during the No-rescue condition (and not a decrease in the Rescue condition, in relation to the baseline).

**Figure 6:**
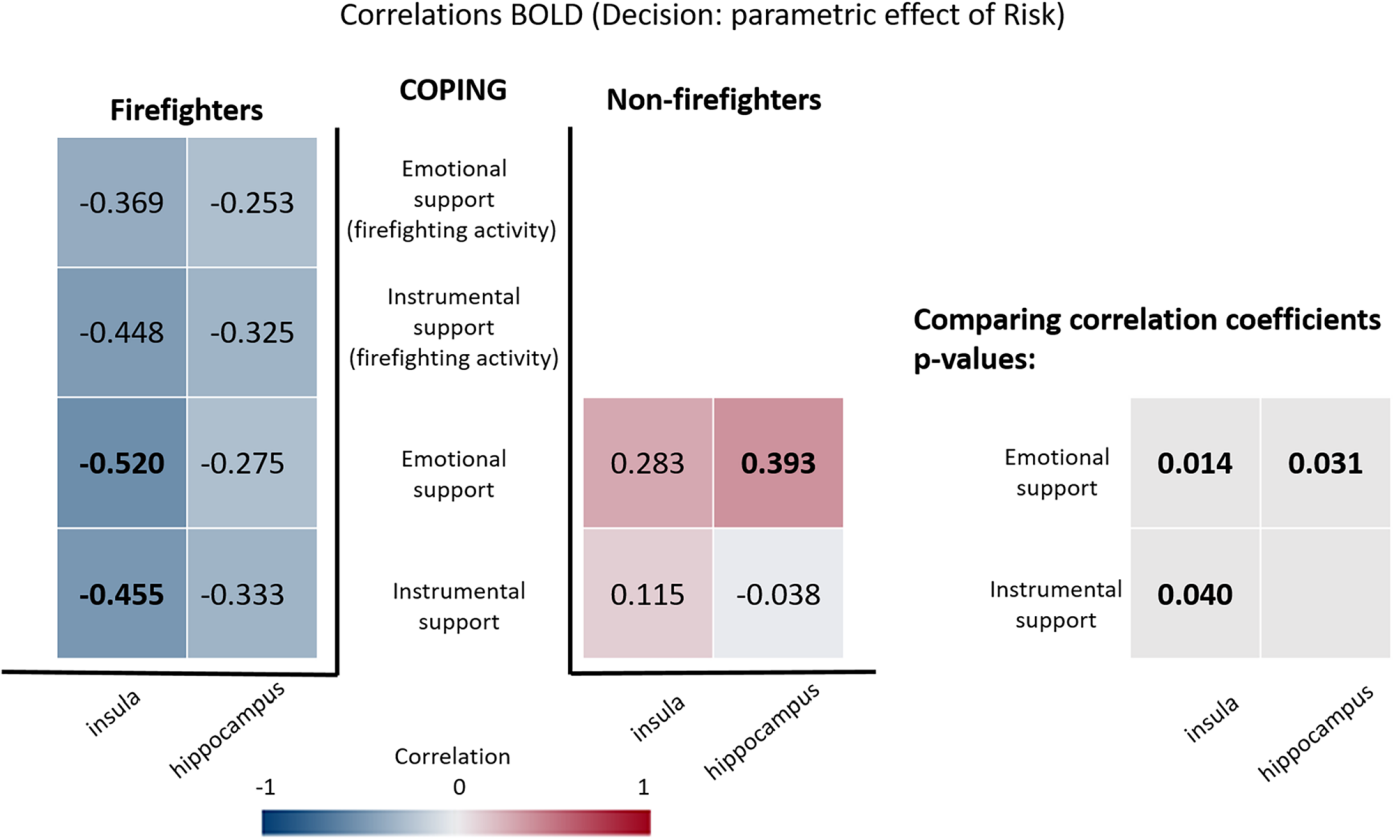
Correlations between individual beta-values from the parametric contrast, in which the decision predictor is modulated by the risk of house collapse, in regions of interest (horizontal axis) and the Emotional and Instrumental strategies of Brief-COPE inventory (vertical axis). Significant correlations are shown in bold. The correlation coefficients are colour coded for visualization purposes (blue for negative and red for positive correlation coefficients). The FDR corrected *p*-values for the comparison of two correlations are presented in the right side and significant values are marked in bold.

Considering the parametric effect of risk, firefighters showed a negative correlation between the beta-values in the insula and the emotional support strategy, while non-firefighters showed a positive correlation in the right hippocampus. The comparison of correlation coefficients, after FDR correction, showed that the correlations are different for emotional support in the insula (Fisher's z = − 2.687, p = 0.014 FDR corrected) and in the right hippocampus (z = − 2.162, p = 0.031 FDR corrected), and for the instrumental support in the insula (z = − 1.879, p = 0.040 FDR corrected) (see Fig. 6).

Supplementary Fig. 7 show a graphical composition that explore the significant correlations between beta-values from the parametric predictor (in which the decision predictor is modulated by the risk of house collapse) and the Emotional and Instrumental strategies of Brief-COPE inventory in the insula.

**Figure 7:**
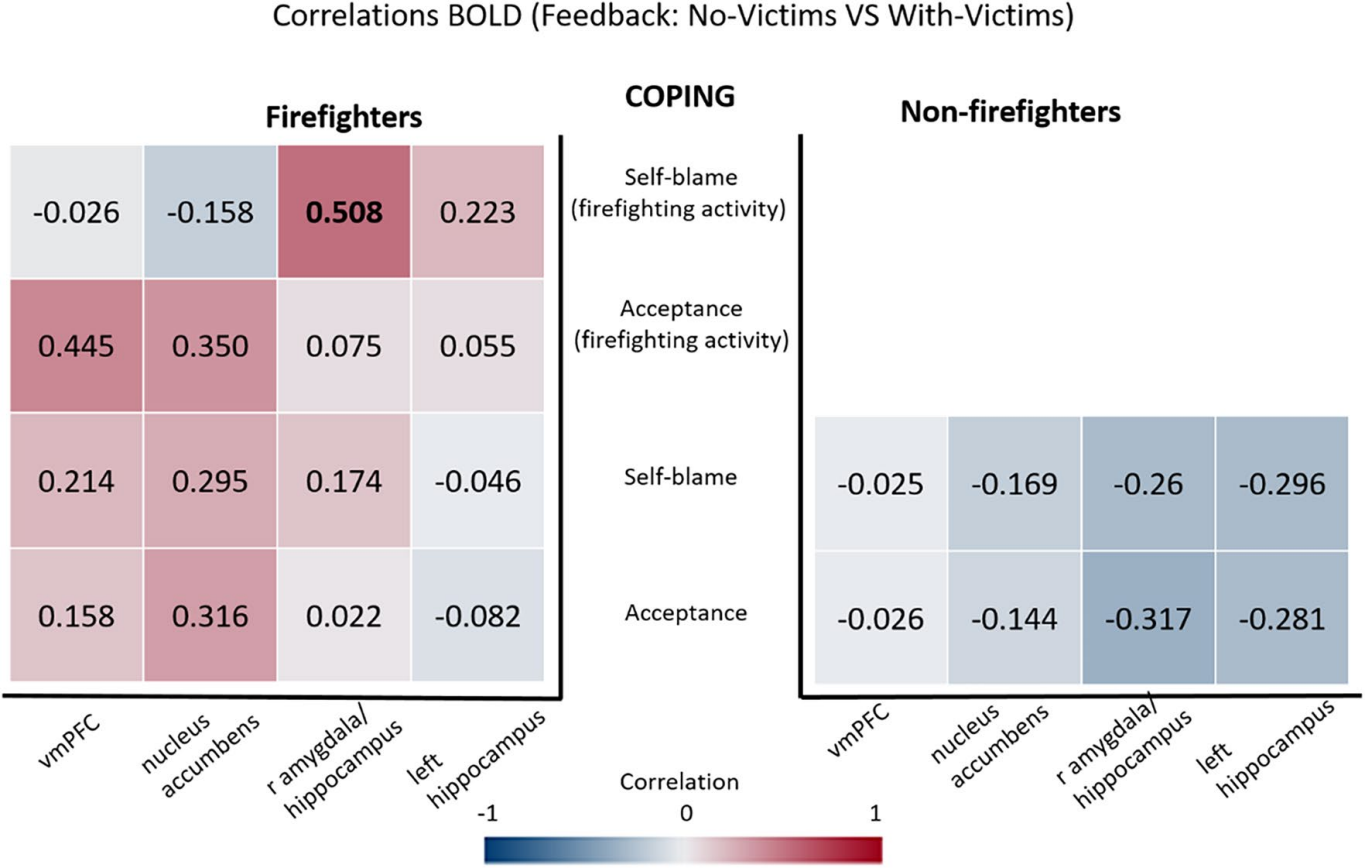
Correlations between individual beta-values from the contrast No-Victims VS Victims in regions of interest (horizontal axis) and the Self-blame and Acceptance strategies of Brief-COPE inventory (vertical axis). Significant correlation is shown in bold. The correlation coefficients are colour coded for visualization purposes (blue for negative and red for positive correlation coefficients).

Considering the feedback phase and the contrast No-victims VS With-victims, firefighters only showed a positive correlation between the beta-values in the amygdala and the score for the self-blame strategy reported in the inventory related to firefighting activity (see Fig. 7). No significant correlations were found within groups for the comparable inventory.

Supplementary Fig. 8 show a graphical composition that explore the significant correlation between individual beta-values from the contrast No-Victims VS With-Victims and the Self-Blame score of Brief-COPE inventory for firefighting activity in the right amygdala/hippocampus. Note that no between groups comparison was done because this correlation refers to a strategy from the Brief-COPE inventory for firefighting activity (the non-firefighters did not respond to that).

For the individual beta-values extracted in these ROIs we found no correlation with PLC-5 score, which is unsurprising given that participants did not score for PTSD in general.

**Figure 8:**
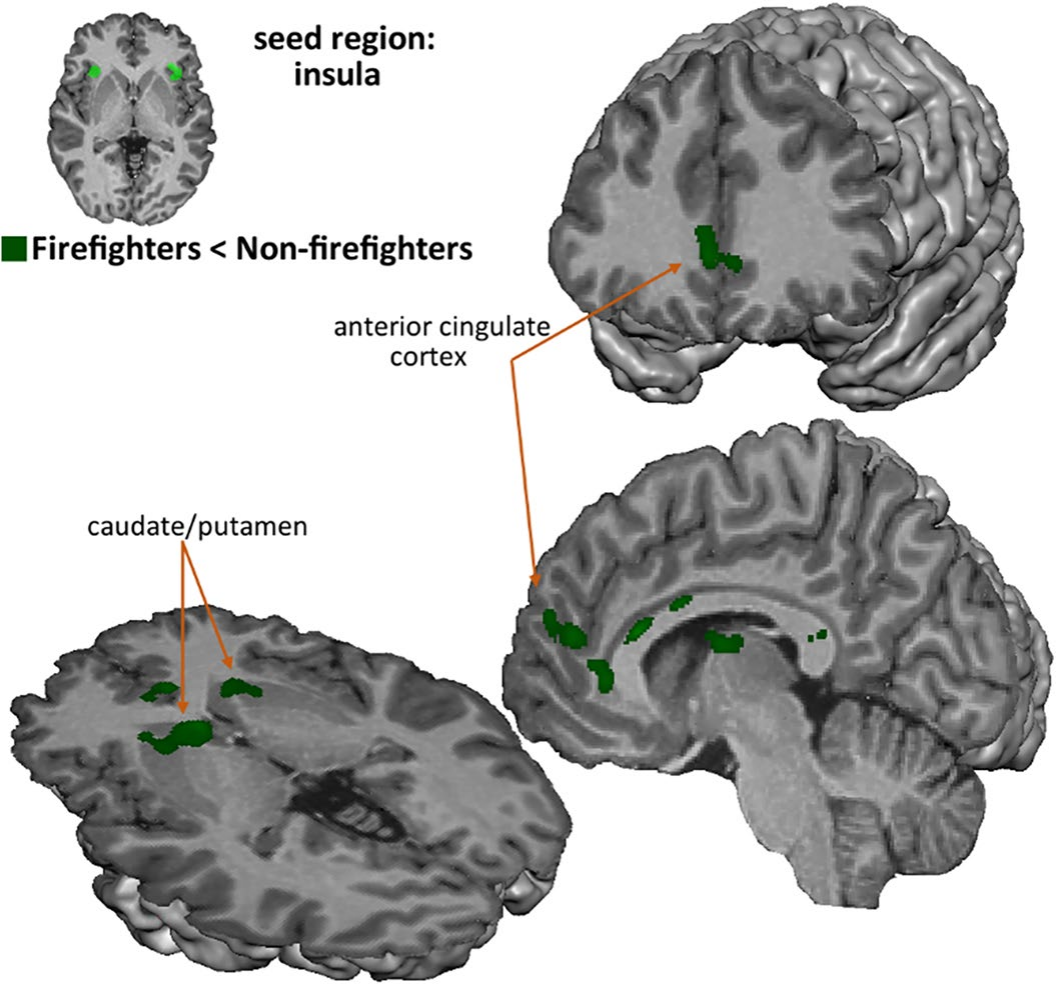
Firefighters versus non-firefighters comparison of effective connectivity from the insula (RFX, t_(46)_ < − 2.69, p < 0.01, minimum cluster size of 57 contiguous voxels). When compared with non-firefighters, the group of firefighters showed decreased influence from insula in the caudate nucleus, putamen, and thalamus, in the anterior and posterior cingulate cortices and in the middle temporal gyrus.

The Granger Causality Mapping (Fig. 8) showed that the insula influenced the activity in the bilateral caudate, putamen, hippocampus, the bilateral inferior frontal and the middle temporal gyri, the cingulate and the ventromedial prefrontal cortices. In sum influences were present on other regions of the salience and affective decision-making networks. Effective connectivity of individual maps was compared between firefighters and non-firefighters. The group of firefighters showed decreased influence from the insula in the caudate nucleus, putamen, and thalamus, in the anterior and posterior cingulate cortices and in the middle temporal gyrus (RFX, t(45) < − 2.69, p < 0.01, minimum cluster size of 57 contiguous voxels).

## Discussion

In this work we tested two main research questions: (1) what are the neural correlates of genuine dilemmas involving self-relevant life-saving decisions, using more realistic firefighting situations; and (2) does expertise change these neural activation patterns at the decision and/or emotional levels? We found evidence that the general-domain system for decision-making also drives decision in the present paradigm involving deontological values. Concerning expertise, we found differences specifically at the behavioral and connectivity levels. We found that expertise does not change activation patterns in the general-domain system involved in rescuing decisions. Importantly, however, we found that expertise changes the effective connectivity patterns, with expert firefighters relying less on directed influences from the insula, and that coping strategies modulate brain activation patterns specifically in firefighters, in particular under rescuing decisions.

The present task involves the consideration of multiple decision variables, which is typical in more realistic tasks. The paradigm was designed with important inputs from experienced firefighters, which resulted in a task capable to engage the participants. Behavioural results showed evidence for this validity, with firefighters deciding faster in particular for non rescuing decisions than non experts. Moreover, participants decided in general to rescue on fewer trials when the risk of the house to collapse was higher. Also, it was interesting to note that participants took the risk more often if the victims were a family instead of only a single adult. In line with this, longer response times were found when participants decided not to rescue, showing differences in the cognitive processes that may be related to make the decision that might violate social expectations.

### A relation between life threatening and general domain decision-making

The task developed in this study involves rapidly assessing the situation (risk of house collapse), identifying potential hazards (number of victims and probability of rescue of the victims by themselves), and determining the appropriate action (enter to rescue or not). It covers strategic decision-making which involves rational reasoning, risk perception and emotional control. Our participants used the probability of house collapse and the number of victims inside the house to decide or not enter in the house. Several studies have varied the probability and magnitude of positive and/or negative outcomes in the neuroeconomic domain, and one in self-irrelevant moral decision-making^4^. Here we introduced a more realistic decision-making paradigm where choice had a self-relevant impact. It involves a trade-off between probability of a burning house collapsing and rescuing probability, in naïve (non-firefighters) and expert decision-makers. Interestingly, our participants took longer to decide when the decision was not to enter in the house, not helping the victims, than when compared with the decision of enter to rescue.

We found evidence that the general-domain system for neuroeconomic decision-making also drives decision in the present paradigm. For instance, comparing the decision of refrain from entering the house for a rescue versus enter to rescue showed higher BOLD activity in the ventral insula, ventromedial prefrontal cortex, caudate head, anterior cingulate cortex and the superior frontal gyrus.

The caudate nucleus is known to connect with several networks playing a role in different emotional and cognitive processes, as reward processing and learning, for goal-directed behaviour^52,53^. It has a role in controlled action selection and inhibition^54–56^, which is in accordance with its activation upon the decision of not entering the house (not helping the victims because of the risk of house collapse). This region is an integral component of cortico-basal ganglia-thalamo-cortical loops, which includes receiving projections form the prefrontal cortex, including the dorsolateral prefrontal cortex and the ventromedial prefrontal cortex^57,58^. In the present study, the ventromedial prefrontal cortex showed relevant modulation during both decision and outcome phases. This region has been associated with the encoding and storage of reward value^52,59–61^. Here, we found that vmPFC to activate more during the decision of enter to rescue than the decision of do not enter. In the feedback phase, vmPFC activates more when there are no victims. The modulation of vmPFC during the outcome phase may be in this case more relevant to the outcome itself rather than a value update. The ventral striatum showed strong activation for the No-Victims VS Victims contrast, in line with the notion that such abstract values as the reward value of human life are also encoded in this subcortical region^59,61–64^.

This task highlighted an important pattern for the anterior insula: it increased activity as a function of the probability of house collapse and increased activity for the decision to withhold rescue, in contrast with the feedback period, during which relevant recruitment in limbic, in particular the amygdala, and reward networks was observed. The modulatory effect, observed while using a parametric predictor of the risk of house collapse**,** allows us to identify regions in which activity increases as the risk increases. The results may reflect both contextual relevance of the stimuli and the decision process. This view is consistent with the generally accepted notion that the anterior insula highlights the most relevant stimuli in order to guide behaviour as part of the salience network^28^. Haufler et al. showed that anterior insula generates bursts of beta oscillations, in which the amplitude reflects the salience of outcomes^27^. The increased activation of anterior insula when participants decided not to rescue may reflect its roles in a variety of functions, namely risk assessment and emotional processing, which are crucial for decision-making^33^. The pattern of activation may also evidence the role of anterior insula in social cognition processing, namely by recognizing social norms and expectations^32^, as the decision of “not to rescue” might violate social expectations. This cognitive processing may be reflected in the slowing of response times for the condition “not to rescue” when compared with the condition “enter to rescue”.

### The role of expertise

The main goal of the present study was to explore how the expertise change the the neurobehavioral correlates of rescuing decisions. Given the relatively high load of training and prior exposure to real rescuing decisions, inner decision models are likely to differ in firefighters. Their expertise is developed through repeated exposure to similar situations that help them recognize patterns and anticipate potential outcomes. Moreover they are highly trained in the contextual use of information (gathering cues from the environment to make sense of the situation and determine the best course of action. This explains why they were faster in deciding, in particular when the option was not to rescue. Interestingly, expertise led to more pronounced changes in connectivity than activation in the domain-general valuation system^67^. Accordingly, considering the effective connectivity from an insular seed region, the group of firefighters showed decreased influence from the insula in striatal regions and to the anterior and posterior cingulate cortex when compared with non-firefighters. As measured by Granger causality analysis, the insula’s connectivity patterns were more pronounced in non-firefighters, suggesting that lack of expertise requires more directed influences from the insula.

Behaviourally, firefighters responded faster than non-firefighters when the decision was “not to rescue”, while both groups responded faster when the decision was to rescue comparing than when it was not to rescue, suggesting a larger social norm conflict in the latter case.

Experts have the opportunity to enhance their coping dimensions with training. We found for firefighter experts a negative correlation between insula activity for the No-rescue VS Rescue contrast and coping dimensions in particular for emotional support (e.g. positive correlation for the opposite contrast). The same held true for the parametric effect of risk. This relation between brain activity and the usage of coping strategies in experts is a relevant finding. We therefore observed a negative correlation between the indexes of neural activity when firefighters decided not to rescue the victims and emotional support and instrumental support facets of coping. By investigating the individual beta-values from this contrast, we conclude that their value comes from an increased activity during the decision of not enter to rescue, rather than a decreased activity during the decision of enter to rescue. This means that the higher the reported usage of coping strategies, the lower the relative neural activity associated to decision of not to rescue (see scatterplots in Supplementary Material). This was found for the insula and caudate nucleus. The same held true for the parametric effect of risk in the insula and right hippocampus. The analysis shows that the usage of coping strategies in daily life (including firefighting activities) is correlated with responsiveness of some regions in firefighters. This pattern was not found in non-firefighters, suggesting that expertise is instrumental in coping mechanisms. This finding may reflect neural adjustment caused by training and/or intensive exposure and repeated exposure which goes hand in hand with the development of coping strategies. It remains an open question whether this difference reflects the concept of neural efficiency which refers to a theoretical construct that comes from the common finding that if individuals become more proficient in a task due to training, they may require fewer cognitive resources, leading to decreased brain activity in those areas associated with task^65,66^. Since there were no differences in the Brief-COPE scores between groups, we suggest that this finding indicates that the use of coping strategies has a greater impact at the neural level for the firefighters than for non-firefighters. However, one should consider that it may alternatively reflect the autobiographic recall of levels of stress and pressure of intensive exposure experienced by firefighters in their work which recruits coping mechanisms. The result highlights the potential role of coping strategies in modulating neural activity during decision-making, particularly in high-stress occupations such as firefighting.

Interestingly, the amygdala showed a positive correlation between the contrast No-Victims VS Victims**,** during feedback, and the Self-blame facet of coping measures (as measured by the Brief-COPE related with the firefighting activity). In other words, the higher the usage of coping strategies, the higher the difference in neural activity between the No-victims feedback and the With-victims feedback. One should consider that it also may reflect that the outcome feedback results have a greater neural impact for those who also need more coping strategies. Please note that Brief-COPE related with the firefighting activity is only available for the firefighters’ group.

Finally, we speculate that correlations of changes in bold activity with behaviour may reflect mechanisms of plasticity that if maladaptive may eventually lead to post-traumatic stress disorder^67^. This would be consistent with the notion that firefighters are a population at risk for post-traumatic stress disorder and psychopathology^12^.

## Conclusions

We found that life-saving dilemmas activate a general domain dilemma solving system, in particular the ventromedial prefrontal cortex, nucleus accumbens, amygdala. Irrespective of explicitly learned deontological norms. Importantly, expertise changed behavioral connectivity patterns.

Differences from expertise at the behavioral level were found for response times, where firefighters responded faster in particular for the decision of not rescue the victims, when compared with non-firefighters. We also found differences between the groups for the directed connectivity from insula, from where the connectivity patterns were stronger in non-firefighters, suggesting that control from the saliency network is reduced in experts.

We also found that experts show modulation of BOLD activation as a function of behavioural and cognitive coping dimensions, in caudate, insula and anterior cingulate cortex (lower activity for non rescuing decisions corresponding to larger coping). This suggests that expertise modulates neural coping mechanisms in life rescuing dilemmas.

### Supplementary Information

Supplementary Information.

## Data Availability

Data will be made available upon reasonable request to the corresponding author.
