# Supplementary material for: Neural underpinnings of ethical decisions in life and death dilemmas in naïve and expert firefighters
Source: Sci Rep. 2024 Jun 8;14:13222. doi: 10.1038/s41598-024-63469-y (PMC11162493; doi:10.1038/s41598-024-63469-y)
Supplement: Supplementary file 1 — Supplementary Information. [file 41598_2024_63469_MOESM1_ESM.docx]

**Neural underpinnings of ethical decisions in life and death dilemmas in naïve and expert firefighters**

**Supplementary Information**

Supplementary table 1. Regions revealed by the contrast No-Rescue VS Rescue, during the decision phase (RFX, t_(45)_ > 4.16, p < 0.01, FDR-corrected). H, hemisphere. R, right. L, Left. ACC, anterior cingulate cortex.

|  |  | MNI coordinates | | |  |  |  |
| --- | --- | --- | --- | --- | --- | --- | --- |
| **region** | **H** | **x** | **y** | **z** | **t** | **p-value** | **mm^3^** |
| insula | R | 31 | 21 | -11 | 6.30 | <0.000001 | 2521 |
| insula | L | -39 | 19 | -17 | 5.42 | 0.000002 | 2331 |
| ACC/superior frontal gyrus | R,L | 5 | 38 | 37 | 5.85 | 0.000001 | 14017 |
| vmPFC | R,L | 5 | 50 | 6 | 4.62 | 0.000032 | 207 |
| caudate | R | 18 | 13 | -1 | 5.08 | 0.000007 | 789 |
| caudate | R | 9 | 16 | 11 | 4.66 | 0.000029 | 410 |
| caudate | L | -9 | 6 | 3 | 5.02 | 0.000009 | 396 |
| posterior cingulate cortex | R,L | 1 | -13 | 30 | 4.89 | 0.000013 | 873 |
| inferior temporal gyrus | R | 58 | -20 | -24 | 5.22 | 0.000004 | 548 |
| angular gyrus | R | 51 | -63 | 33 | 4.94 | 0.000011 | 696 |

Supplementary table 2. Regions revealed by the parametric contrast modulated by the risk of house collapse (conjunction analysis with the main contrast) during the decision phase (RFX, t_(46)_ > 2.69, p < 0.01, minimum cluster size of 24 contiguous voxels). H, hemisphere. R, right. L, Left. PFC, prefrontal cortex.

|  |  | MNI coordinates | | |  |  |  |
| --- | --- | --- | --- | --- | --- | --- | --- |
| **region** | **H** | **x** | **y** | **z** | **t** | **p-value** | **mm^3^** |
| hippocampus | R | 28 | -26 | -18 | 3.78 | 0.000444 | 1312 |
| anterior insula | R | 38 | 19 | -4 | 4.19 | 0.000123 | 1886 |
| anterior insula | L | -35 | 23 | -2 | 4.62 | 0.000031 | 1037 |
| dorsolateral PFC | R | 41 | 23 | 23 | 3.16 | 0.002756 | 295 |
| medial frontal gyrus | R,L | -1 | 19 | 48 | 4.71 | 0.000023 | 5220 |
| posterior cingulate cortex | R | 20 | -55 | 14 | 3.88 | 0.000331 | 629 |
| posterior cingulate cortex | R,L | 0 | -32 | 33 | 4.05 | 0.000197 | 3376 |
| midbrain | R,L | 1 | -25 | -3 | 4.55 | 0.000039 | 1320 |
| inferior occipital gyrus | R | 45 | -79 | -11 | 3.28 | 0.00201 | 328 |
| fusiform gyrus | R | 43 | -49 | -20 | 3.29 | 0.001952 | 299 |
| inferior parietal lobule BA40 | R | 44 | -57 | 54 | 3.00 | 0.004306 | 234 |

Supplementary table 3. Regions revealed by the contrast No-Victims VS With-victims during the feedback phase (RFX, -3.42 > t_(46)_ > 3.42, p < 0.01, FDR-corrected). H, hemisphere. R, right. L, Left. PFC, prefrontal cortex.

|  |  | MNI coordinates | | |  |  |  |
| --- | --- | --- | --- | --- | --- | --- | --- |
| **region** | **H** | **x** | **y** | **z** | **t** | **p-value** | **mm^3^** |
| nucleus accumbens | R | 11 | 11 | -8 | 5.36 | 0.000003 | 775 |
| nucleus accumbens | L | -11 | 8 | -8 | 4.21 | 0.000116 | 199 |
| amygdala anterior hippocampus | R | 33 | -9 | -20 | 6.25 | <0.000001 | 6214 |
| hippocampus | L | -22 | -16 | -16 | 5.24 | 0.000004 | 1160 |
| ventromedial PFC | R, L | 0 | 55 | -6 | 5.41 | 0.000002 | 1936 |
| cingulate gyrus BA24 | R, L | -4 | -5 | 51 | 6.46 | <0.000001 | 3350 |
| dorsolateral PFC | L | -31 | 38 | 39 | 4.06 | 0.000191 | 287 |
| temporal pole | R | 37 | 10 | -23 | 4.91 | 0.000012 | 237 |
| postcentral gyrus | R | 28 | -42 | 65 | 4.19 | 0.000123 | 414 |
| postcentral gyrus | L | -5 | -36 | 75 | 4.37 | 0.000071 | 326 |
| precentral gyrus | L | -39 | -26 | 46 | 7.77 | <0.000001 | 37877 |
| occipital cortex | R, L | 28 | -94 | 2 | 9.37 | <0.000001 | 126654 |
| intraparietal sulcus | R | 24 | -71 | 44 | -5.24 | 0.000004 | 1430 |
| intraparietal sulcus | L | -22 | -70 | 37 | -3.86 | 0.000356 | 212 |
| caudate | L | -10 | 8 | 9 | -4.45 | 0.000054 | 298 |
| parahippocampal gyrus | R | 29 | -55 | -7 | -4.80 | 0.000017 | 734 |
| parahippocampal gyrus | L | -29 | -56 | -8 | -5.00 | 0.000009 | 778 |
| supramarginal gyrus BA40 | R | 54 | -53 | 27 | -4.88 | 0.000013 | 1316 |
| supramarginal gyrus BA40 | L | -50 | -56 | 30 | -5.26 | 0.000004 | 2405 |
| insula | R | 32 | 22 | -15 | -5.71 | 0.000001 | 3255 |
| insula | L | -54 | 22 | 6 | -5.97 | <0.000001 | 11814 |
| middle frontal gyrus | R | 42 | 21 | 53 | -4.92 | 0.000012 | 432 |
| middle frontal gyrus | L | -37 | 18 | 44 | -6.24 | <0.000001 | 4926 |
| medial frontal gyrus | R, L | -4 | 27 | 55 | -7.09 | <0.000001 | 17502 |
| temporal pole | L | -46 | 15 | -42 | -5.41 | 0.000002 | 1400 |


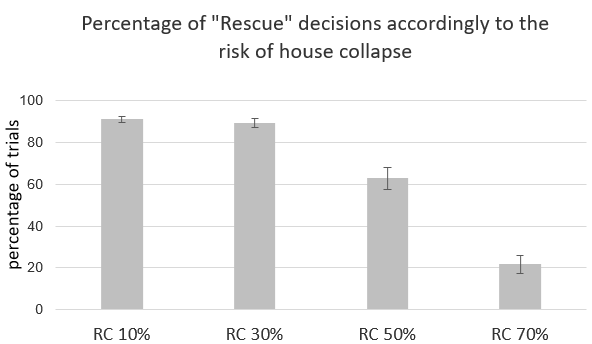


Supplementary Figure 1: Percentage of “Rescue” decisions considering the risk of the house collapse that could be 10%, 30%, 50%, or 70%. Participants decided to enter in the house to rescue the victims in less trails when the risk of collapse was higher.


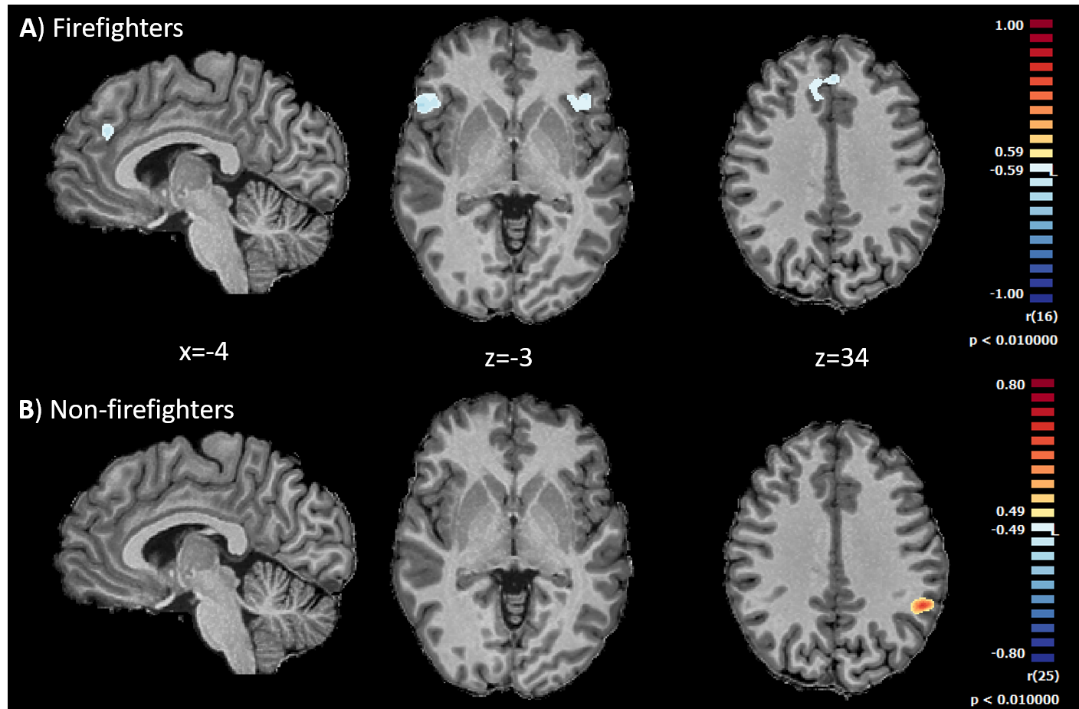


Supplementary Figure 2: Whole-brain correlations between individual beta-values from the contrast No-rescue VS Rescue and the Emotional Support strategies of Brief-COPE inventory. Panel A shows this correlation for the Firefighters (r(16) < -0.59, p<0.01 non-corrected, minimum cluster size of 75 contiguous voxels, manually set), while panel B shows the correlation for the Non-firefighters group (r(25) < -0.49, p<0.01 non-corrected, minimum cluster size of 75 contiguous voxels, manually set). Blue colour mark negative correlation coefficients, while red for positive correlation coefficients. Supplementary table 4 lists all clusters.

Supplementary table 4. Regions from hole-brain correlations between individual beta-values from the contrast No-rescue VS Rescue and the Emotional Support strategies of Brief-COPE inventory (p<0.01 non-corrected, minimum cluster size of 75 contiguous voxels, manually set). H, hemisphere. R, right. L, Left. ACC, anterior cingulate cortex.

|  |  | MNI coordinates | | |  | cluster stats | |
| --- | --- | --- | --- | --- | --- | --- | --- |
| region | **H** | **x** | **y** | **z** | **mm^3^** | **r** | **p-value** |
| Firefighters |  |  |  |  |  |  |  |
| insula | R | 53 | 22 | 2 | 2288 | -0.73 | 0.000591 |
| insula | L | -40 | 25 | -2 | 947 | -0.70 | 0.001263 |
| anterior cingulated cortex | R,L | 2 | 35 | 32 | 651 | -0.64 | 0.004153 |
| Non-firefighters |  |  |  |  |  |  |  |
| inferior temporal gyrus | R | 65 | -19 | -25 | 875 | 0.57 | 0.001707 |
| angular gyrus | L | -48 | -47 | 42 | 2228 | 0.63 | 0.000411 |


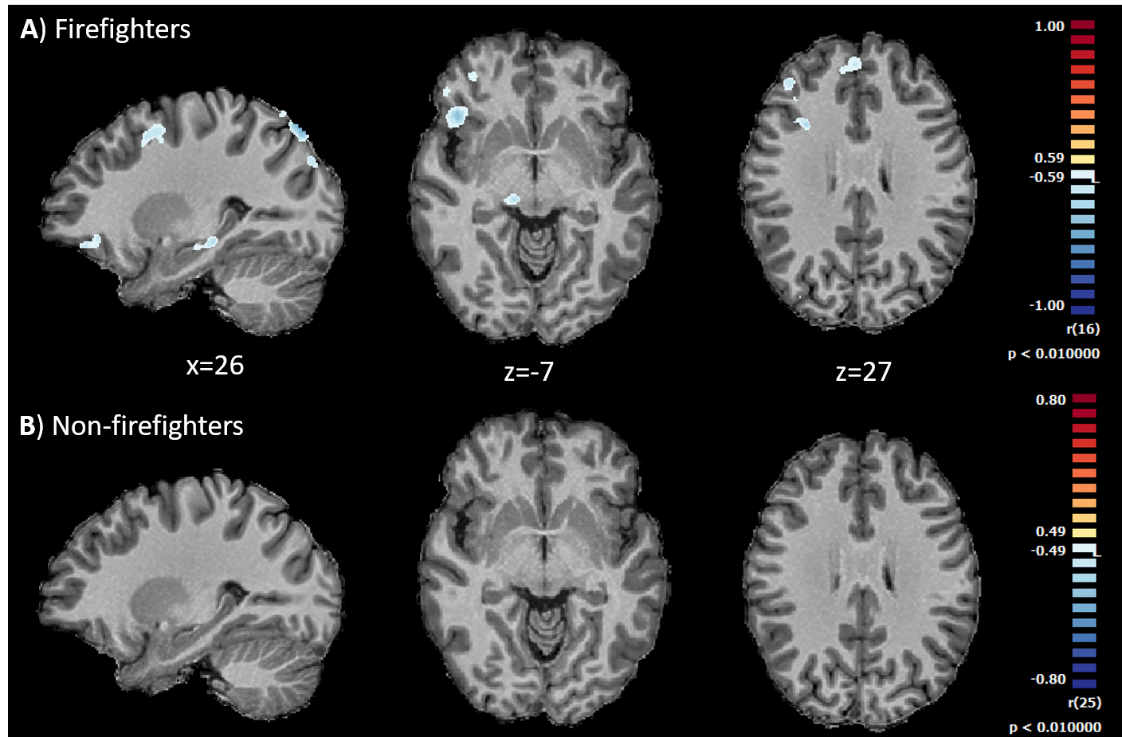


Supplementary Figure 3: Whole-brain correlations between individual beta-values from the contrast No-rescue VS Rescue and the Instrumental Support strategies of Brief-COPE inventory. Panel A shows this correlation for the Firefighters (r(16) < -0.59, p<0.01 non-corrected, minimum cluster size of 75 contiguous voxels, manually set), while panel B shows the correlation for the Non-firefighters group (r(25) < -0.49, p<0.01 non-corrected, minimum cluster size of 75 contiguous voxels, manually set). Blue colour mark negative correlation coefficients. Supplementary table 5 lists all clusters.

Supplementary table 5. Regions from whole-brain correlations between individual beta-values from the contrast No-rescue VS Rescue and the Instrumental Support strategies of Brief-COPE inventory (p<0.01 non-corrected, minimum cluster size of 75 contiguous voxels, manually set). H, hemisphere. R, right. L, Left. ACC, anterior cingulate cortex.

|  |  | MNI coordinates | | |  | cluster stats | |
| --- | --- | --- | --- | --- | --- | --- | --- |
| **region** | **H** | **x** | **y** | **z** | **mm^3^** | **r** | **p-value** |
| **Firefighters** |  |  |  |  |  |  |  |
| medial PFC | R,L | 5 | 48 | 27 | 716 | -0.64 | 0.003999 |
| dorsolateral PFC | R | 39 | 33 | 19 | 4131 | -0.80 | 0.000075 |
| pars opercularis (BA44) | R | 57 | 20 | 14 | 2213 | -0.79 | 0.000082 |
| pars orbitalis (BA 47) | R | 41 | 33 | -12 | 3820 | -0.77 | 0.000169 |
| supplementary motor area (BA6) | R | 28 | 8 | 49 | 1000 | -0.82 | 0.000037 |
| precuneus (BA 7) | R | 35 | -80 | 37 | 1354 | -0.82 | 0.000033 |
| precuneus (BA 7) | R | 21 | -79 | 49 | 2422 | -0.84 | 0.000011 |
| precuneus (BA 7) | L | -23 | -70 | 59 | 1133 | -0.75 | 0.000311 |
| precuneus (BA 7) | L | -28 | -81 | 47 | 1966 | -0.80 | 0.000056 |
| midbrain | R | 15 | -26 | -12 | 1378 | -0.79 | 0.000104 |
| cerebellum | L | -23 | -57 | -17 | 994 | -0.73 | 0.000524 |
| cerebellum | L | 1 | -74 | -19 | 693 | -0.77 | 0.000166 |
| **Non-firefighters** |  |  |  |  |  |  |  |
| -- |  |  |  |  |  |  |  |


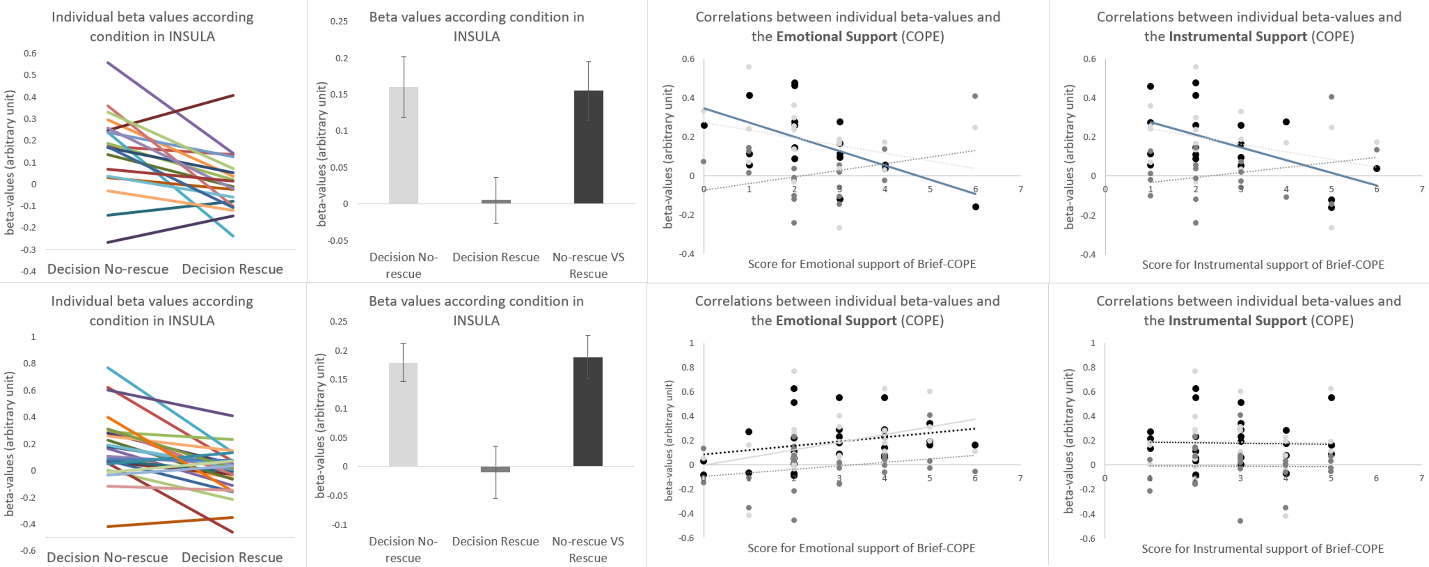


Supplementary Figure 4: Graphical composition to explore significant correlations between individual beta-values from the contrast No-rescue VS Rescue and the Emotional and Instrumental strategies of Brief-COPE inventory in the insula. Top line for Firefighters group, and bottom line for Non-firefighters. First column shows the individual beta-values for the conditions No-rescue and Rescue. Second column shows the mean and the standard error of the mean of the beta-values. Scatterplots and linear trends show the relations between beta-values of the contrast No-rescue VS Rescue (black) or No-rescue (light grey) or Rescue (grey) and the scores of the Emotional and Instrumental strategies (bold trends show significant correlations). Comparisons between group coefficients were only made for the correlations between the contrast No-rescue VS Rescue and the coping strategy. Note that the correlations for the Emotional support were significantly different between groups (p=0.008, FDR corrected).


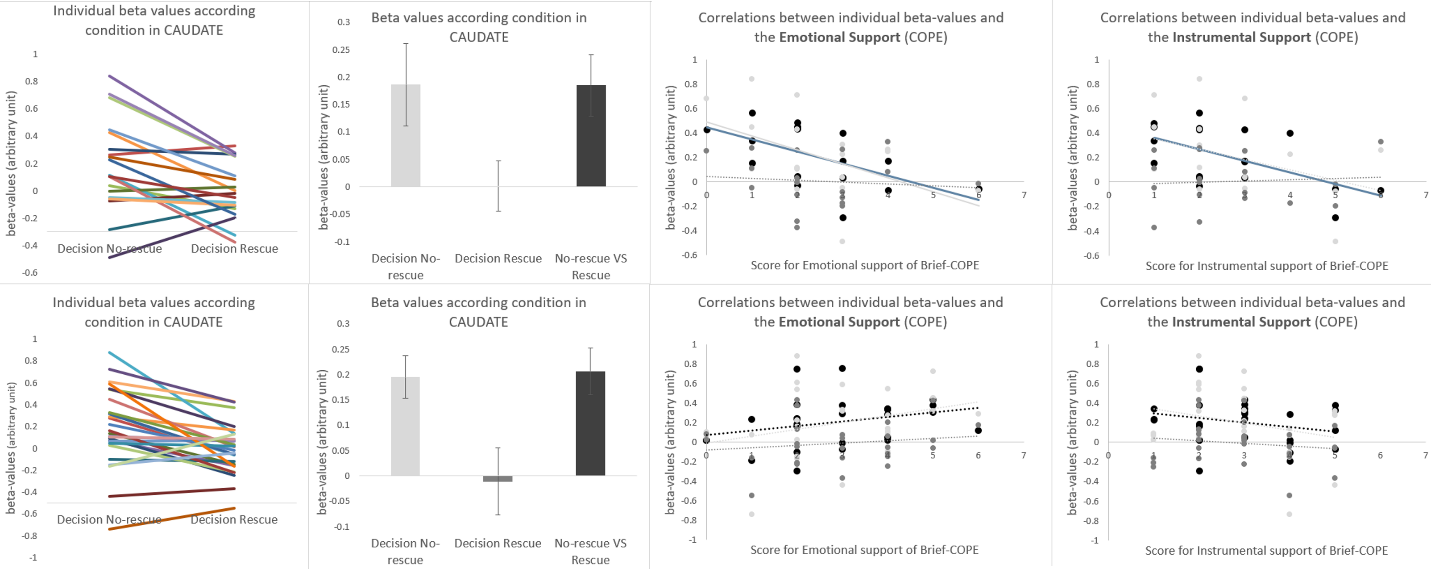


Supplementary Figure 5: Graphical composition to explore significant correlations between individual beta-values from the contrast No-rescue VS Rescue and the Emotional and Instrumental strategies of Brief-COPE inventory in the caudate nucleus. Top line for Firefighters group, and bottom line for Non-firefighters. First column shows the individual beta-values for the conditions No-rescue and Rescue. Second column shows the mean and the standard error of the mean of the beta-values. Scatterplots and linear trend show the relation between beta-values of the contrast No-rescue VS Rescue (black) or No-rescue (light grey) or Rescue (grey) and the scores of the Emotional and Instrumental strategies (bold trends show significant correlations). Comparisons between group coefficients were only made for the correlations between the contrast No-rescue VS Rescue and the coping strategy. Note that the correlations for the Emotional support were significantly different between groups (p=0.008, FDR corrected).


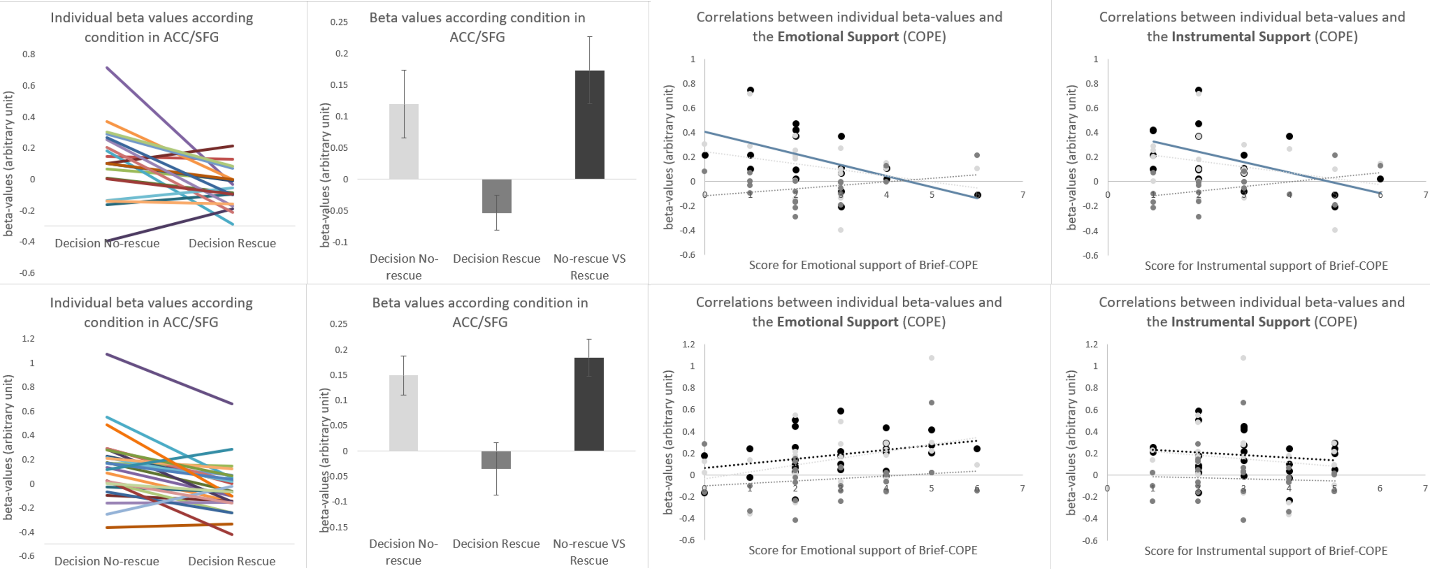


Supplementary Figure 6: Graphical composition to explore significant correlations between individual beta-values from the contrast No-rescue VS Rescue and the Emotional and Instrumental strategies of Brief-COPE inventory in the anterior cingulate cortex/superior frontal gyrus. Top line for Firefighters group, and bottom line for Non-firefighters. First column shows the individual beta-values for the conditions No-rescue and Rescue. Second column shows the mean and the standard error of the mean of the beta-values. Scatterplots and linear trend show the relation between beta-values of the contrast No-rescue VS Rescue (black) or No-rescue (light grey) or Rescue (grey) and the scores of the Emotional and Instrumental strategies (bold trends show significant correlations). Comparisons between group coefficients were only made for the correlations between the contrast No-rescue VS Rescue and the coping strategy. Note that the correlations for the Emotional support were significantly different between groups (p=0.043, FDR corrected).


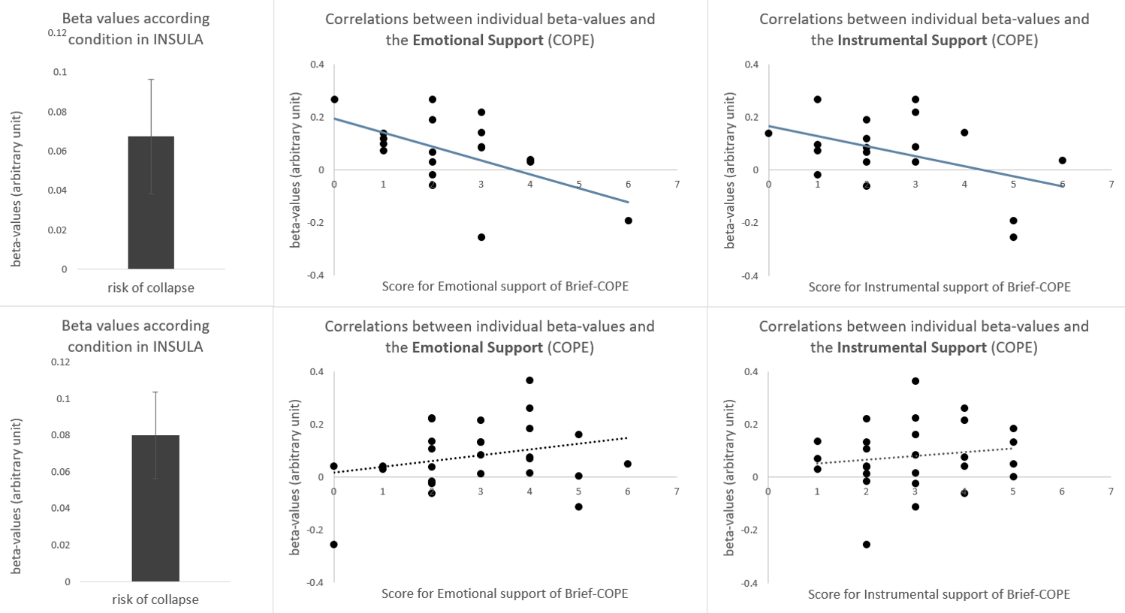


Supplementary Figure 7: Graphical composition to explore significant correlations between individual beta-values from the parametric predictor, in which the decision predictor is modulated by the risk of house collapse, and the Emotional and Instrumental strategies of Brief-COPE inventory in the insula. Top line for Firefighters group, and bottom line for Non-firefighters. First column shows the mean and the standard error of the mean of the beta-values of the parametric predictor. Scatterplots and linear trend show the relation between beta-values and the scores of the Emotional and Instrumental strategies (bold trends show significant correlations). Note that the correlations for the Emotional support and for the Instrumental support were significantly different between groups (p=0.014 and p=0.040, FDR corrected).


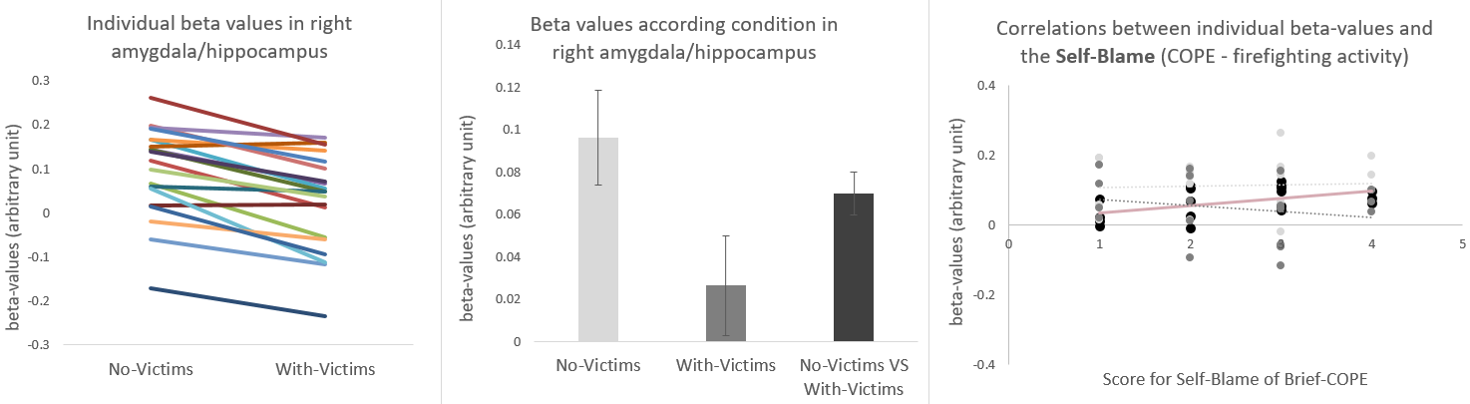


Supplementary Figure 8: Graphical composition to explore the significant correlation between individual beta-values from the contrast No-Victims VS With-Victims and the Self-Blame score of Brief-COPE inventory for firefighting activity in the right amygdala/hippocampus. Only Firefighters group is presented because it refers to the Brief-COPE inventory for firefighting activity. First column shows the individual beta-values for the conditions No-Victims and With-Victims. Second column shows the mean and the standard error of the mean of the beta-values. Scatterplot and linear trend show the relation between beta-values of the contrast No-Victims VS With-Victims (black) or No-Victims (light grey) or No-Victims (grey) and the scores of the Self-Blame strategy (bold trend show significant correlation).
